# Xenorhodopsins, an enigmatic new class of microbial rhodopsins horizontally transferred between archaea and bacteria

**DOI:** 10.1186/1745-6150-6-52

**Published:** 2011-10-10

**Authors:** Juan A Ugalde, Sheila Podell, Priya Narasingarao, Eric E Allen

**Affiliations:** 1 Marine Biology Research Division, Scripps Institution of Oceanography, University of California, San Diego, La Jolla, CA 92093-0202, USA; 2 Division of Biological Sciences, University of California, San Diego, La Jolla, CA 92093, USA

## Abstract

Based on unique, coherent properties of phylogenetic analysis, key amino acid substitutions and structural modeling, we have identified a new class of unusual microbial rhodopsins related to the Anabaena sensory rhodopsin (ASR) protein, including multiple homologs not previously recognized. We propose the name xenorhodopsin for this class, reflecting a taxonomically diverse membership spanning five different Bacterial phyla as well as the Euryarchaeotal class Nanohaloarchaea. The patchy phylogenetic distribution of xenorhodopsin homologs is consistent with historical dissemination through horizontal gene transfer. Shared characteristics of xenorhodopsin-containing microbes include the absence of flagellar motility and isolation from high light habitats.

Reviewers: This article was reviewed by Dr. Michael Galperin and Dr. Rob Knight.

## Findings

Microbial rhodopsins are a widespread family of photoactive proteins found in all three domains of life. Based on their functional roles, characterized rhodopsin proteins have been classified into three distinct groups: (i) Proton pumps (bacteriorhodopsins and proteorhodopsins), involved in energy generation, (ii) Chloride pumps (halorhodopsins), involved in the maintenance of osmotic balance, and (iii) Sensory rhodopsins, which direct positive and/or negative phototaxis. Microbial proton pumps have the widest ecological niche distribution, and are found throughout the Bacteria and Archaea in hypersaline, marine, and freshwater habitats [1]. Chloride pumps and sensory rhodopsins are mostly limited to halophilic Archaea of class Halobacteria [1], excepting the few characterized examples in the freshwater cyanobacterium *Anabaena (Nostoc) *sp. PCC 7120 [2,3] and eukaryotic green algae including *Chlamydomonas reinhardtii *[4].

The evolutionary history of microbial rhodopsins is complex, showing broad but patchy phylogenetic distribution within and across disparate lineages. It has been suggested that horizontal gene transfer (HGT) has disseminated photoreceptor and photosensory activities across large evolutionary distances [1]. One salient example is a putative sensory rhodopsin found in the bacterium *Anabaena (Nostoc) *sp. PCC 7120 (Anabaena sensory rhodopsin, ASR). It has been suggested that this protein was originally acquired from a halophilic archaeon by HGT, and may play a sensory role [1,2]. However, sensory function performance has not yet been demonstrated experimentally, and the ASR protein differs from previously described sensory rhodopsins in: (i) a distinct signaling cascade mechanism that employs a soluble transducer protein, rather than the methyl-accepting taxis transducers (HTR proteins) found in halophilic Archaea [2,5] and (ii) its divergent photochemistry, including unique light-induced *cis/trans *configuration dynamics of the retinal chromophore, providing a possible mechanism for sensing and differentiating specific light qualities [3,6].

In the current study, we report the discovery of several new ASR protein homologs with shared characteristics consistent with the designation of a new class of microbial rhodopsins. ASR homologs were found in *Nanosalina *sp. J07AB43 and *Nanosalinarum *sp. J07AB56, the first representatives of a newly described major lineage of Archaea (class Nanohaloarchaea) within phylum Euryarchaeota [7]. The *Nanosalina *sp. and *Nanosalinarum *sp. rhodopsin proteins are highly similar to each other (89% amino acid identity) and are present in both genomes as single copy genes. Surprisingly, these two Nanohaloarchaeal proteins most closely resemble rhodopsins in taxonomically distant *Cyanothece *sp. PCC 7424 and *Anabaena *(*Nostoc*) sp. PCC 7120, at 31 and 34% amino acid identity respectively. No homologs were identified in other members of the Euryarchaeota, although related proteins were detected at 30-31% amino acid identity in *Bacillus coahuilensis *m4-4 (phylum Firmicutes), a sporulating halophilic bacterium isolated from a desiccation lagoon [8], the psychrophilic bacterium *Hymenobacter roseosalivarius *AA-718 (phylum Bacteroidetes), and the halophilic bacterium *Haloplasma contractile *SSD-17B (phylum Tenericutes) [9,10].

**Figure 1:**
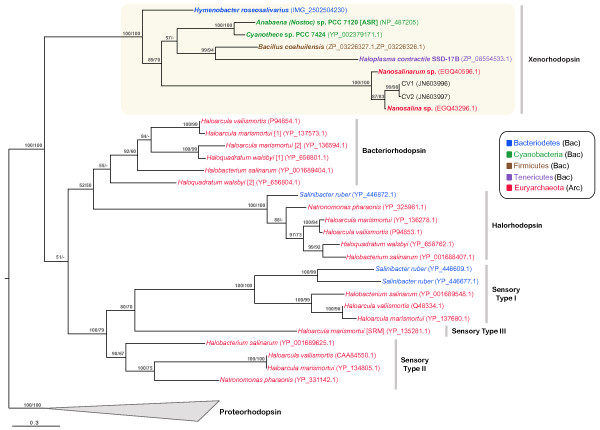
**Phylogenetic tree of microbial rhodopsin proteins showing diversity of functional classes**. Tree is based on a total of 34 sequences (205 amino acid positions) using maximum likelihood and Bayesian inference methods. Numbers at nodes represent posterior probablities inferred by MrBayes (first value) and maximum likelihood bootstrap values using RaxML (second value). Only values greater than 50% are shown. GenBank accession numbers are shown in parentheses for each protein except *H. roseosalivarus *(IMG-ER database gene object ID) [22]. Sequences CV1 and CV2 were recovered by PCR amplification of environmental DNA from a solar saltern in Chula Vista, California, USA, using Nanohaloarchaeal-specific xenorhodopsin primers (see Additional File 1).

Figure 1 shows a phylogenetic analysis using maximum likelihood and Bayesian inference methods for the ASR homologs, together with a set of representative protein sequences from all previously recognized functional microbial rhodopsin classes. Methods and experimental procedures are provided in Additional File 1. The phylogenetic tree also includes additional sequences we obtained by PCR amplification using primers specifically targeting Nanohaloarchaeal rhodopsin genes. These sequences were recovered from a hypersaline environment (South Bay Salt Works, Chula Vista, California, USA) that is geographically distant from the original isolation site of the Nanohaloarchaea genomes (Lake Tyrrell, Victoria, Australia). Tree topology shows robust clustering of all ASR homologs as a single clade, distinct from other rhodopsin types. We propose the name "xenorhodopsins" to describe this class of rhodopsin proteins, articulating the wide taxonomic diversity of its members.

The patchy distribution and topology of the xenorhodopsin clade is consistent with HGT events between domains and involving five disparate bacterial phyla. The large numbers of currently sequenced Firmicute (873), Bacteriodetes (169), Cyanobacteria (68), and Haloarchaea genomes (18) lacking xenorhodopsin homologs make it unlikely that the gene/species tree incongruencies shown in Figure 1 could be explained by independent gene loss among multiple species. Sufficiency of taxon sampling and information content in our 205-position trimmed amino acid sequence alignment (Supplementary File 2) are well supported by significant bootstrap values (Figure 1), and corroborated by complete topological agreement between trees constructed using Bayesian and maximum likelihood methods. Additionally, the new xenorhodopsin sequences identified here do not change overall tree topologies of other microbial rhodopsin sequences previously reported in the literature [11].

To supplement HGT analysis based on phylogenetic incongruencies, DNA signature patterns were analyzed for individual xenorhodopsin proteins relative to the genomes in which they were found, based on percent G+C, codon usage patterns, and Interpolated Variable Order Motifs [12] (Additional File 1: Table S1). By all of these criteria, xenorhodopsin genes in *Nanosalinarum *J07AB56, *Cyanothece *PCC 7424, *Nostoc *PCC 7120, *Hymenobacter roseosalivarius*, and *Haloplasma contractile *closely resemble other loci within their respective genomes. These data support the likelihood that the observed incongruencies between xenorhodopsin protein and species trees for these genomes represent ancient rather than recent HGT events, with subsequent amelioration of foreign DNA signatures over time. A different pattern was observed for xenorhodopsin proteins in the *Bacillus coahuilensis *and *Nanosalina *J07AB43 genomes, where atypical codon usage suggests that HGT events may have occurred more recently (Additional File 1: Table S1).

The absence of xenorhodopsin genes in all Euryarchaeaota other than members of class Nanohaloarchaea suggests that these genes were acquired subsequent to divergence of Nanohaloarchaeaota from other Euryarchaeotal classes. The high degree of similarity among xenorhodopsin proteins obtained from two different Nanohalorchaeal genera, as well as environmental sequences from a distant geographical location (North America versus Australia), is consistent with inheritance from a common ancestral source, coupled with strong selective pressure for amino acid sequence conservation. The discrepancy between ancestral inheritance and the atypical codon usage pattern observed in the *Nanosalina *J07AB43 protein may be explained by relatively recent secondary exchange with other Nanohaloarchaea, as multiple genera of this lineage are known to coexist in shared habitats [7].

The phylogenetic tree presented in Figure 1 includes only known, modern representatives of lineages that may have incorporated multiple HGT events between extinct ancestors and/or serial exchanges with unknown species whose genomes have not yet been sequenced. Although the complexity of these relationships precludes confident reconstruction of the exact timing, direction, and order of individual gene transfer events, cross-domain and cross-phylum gene acquisition through HGT provides the most parsimonious explanation for the data.

**Figure 2:**
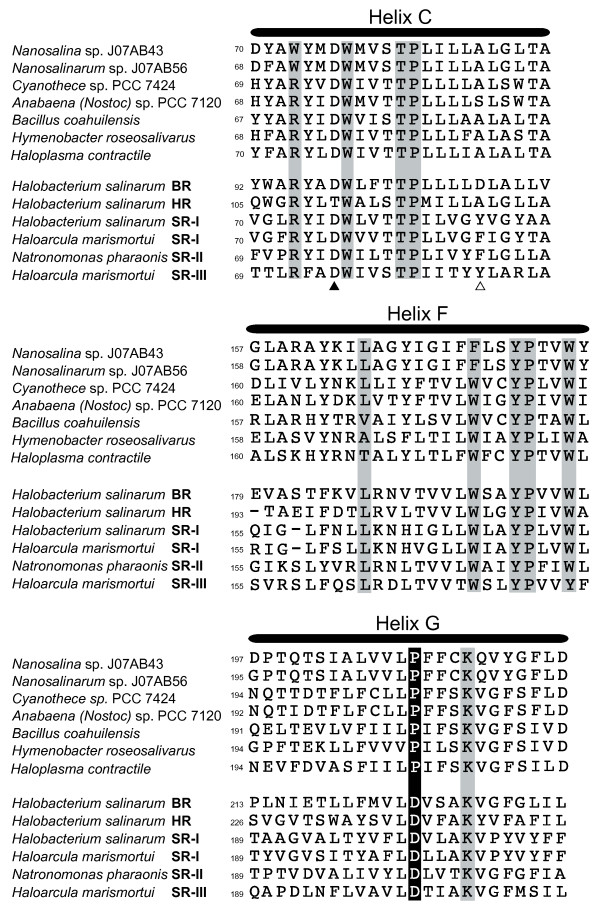
**Protein alignment of key amino acid segments**. Sequences include all xenorhodopsin homologs plus selected representatives of previously characterized microbial rhodopsin functional classes; bacteriorhodopsin (BR), halorhodopsin (HR) and sensory rhodopsins I (SR-I), II (SR-II) and III (SR-III). Shaded boxes indicate conserved residues involved in retinal binding [3]. The black box in Helix G shows a conserved Asp to Pro substitution in all xenorhodopsin proteins at this position. Retinal Schiff base proton acceptor (closed triangle) and proton donor (open triangle) residues are marked in Helix C.

Amino acid alignments of residues known to determine function for previously characterized microbial rhodopsins are inconsistent with proton or chloride transporting activity for xenorhodopsins, suggesting a possible sensory role (see Additional File 2 for full alignment). Figure 2 shows that residues required to bind the retinal chromophore molecule are conserved across all xenorhodopsin group members. Ion transporting rhodopsins can be distinguished from sensory rhodopsins by comparing the residues that serve as the retinal Schiff base proton donor and proton acceptor during the photocycle [2,13]. These residues correspond to Asp98 (acceptor) and Asp109 (donor) in the *H. salinarum *bacteriorhodopsin (Helix C). Consistent with previously described sensory rhodopsins, ASR and all other xenorhodopsin homologs lack the canonical Asp residue at the donor position, a hallmark of proton translocating rhodopsins. Likewise, known sensory rhodopsins and xenorhodopsins both lack the Thr (acceptor) and Ala (donor) configuration diagnostic of chloride pumps (Figure 2).

Despite the insights provided by these results, it is not possible to predict functional activity based on sequence alignment alone. The structural sensitivity of microbial rhodopsins is highlighted by the ability to engineer aberrant functional properties in these proteins. A single amino acid substitution, Asp217 to Glu, has been shown to confer inward proton pumping activity to the ASR protein [14] and a single amino acid substitution is sufficient to convert a bacteriorhodopsin proton pump into a chloride pump [15].

One prominent difference between the xenorhodopsins and all other microbial rhodopsin proteins is a universal Pro to Asp substitution (Helix G), a substitution noted previously in the *Anabaena (Nostoc) *sp. PCC 7120 and *B. coahuilensis *homologs [8,16]. The shared position of this residue in all xenorhodopsins discovered to date suggests that it may provide a useful diagnostic for this protein class.

Sequence conservation and phylogenetic analysis of xenorhodopsin proteins is strongly supported by comparative 3-dimensional protein structure modeling. This similarity is illustrated in Additional File 3, showing a SWISS-MODEL [17] prediction of the *Nanosalina *sp. rhodopsin structure using ASR as a template. The modeled structure demonstrates high congruence in residues that form the retinal binding pocket, as well as similar truncations in loop motifs (Additional File 3). The conserved primary and tertiary structure of xenorhodopsins combined with their distinct phylogenetic clustering supports their classification as a coherent, highly conserved group.

**Figure 3:**
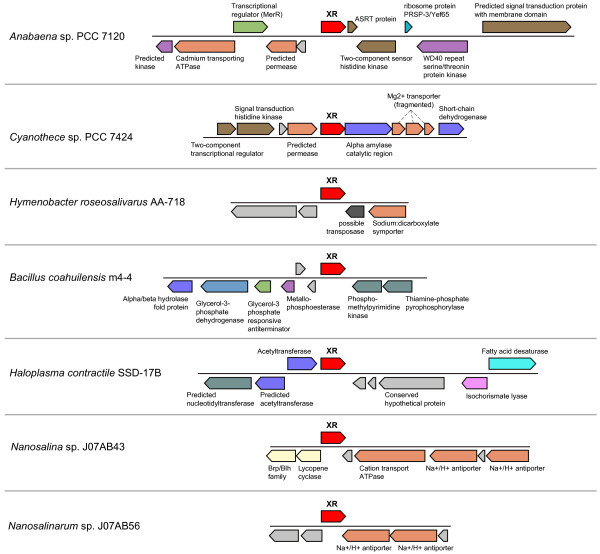
**Genomic neighborhood of xenorhodopsin (XR) genes**. Functional annotations were obtained from NCBI [23] and IMG [22]. Similar gene functions are color-coded. Hypothetical proteins are unlabeled and shown in gray.

An important element of previously characterized sensory rhodopsins in halophilic Archaea is the presence of a signal transduction mechanism, most often genetically encoded in a genomic position adjacent to the rhodopsin gene [18]. In *Anabaena (Nostoc) *sp. PCC 7120, the proposed soluble transducer protein ASRT (*Anabaena *sensory rhodopsin transducer) is encoded by a gene in the same operon as ASR [18] (Figure 3). Consistent with its putative role in light-activated sensory transduction, the ASRT protein has been shown to bind DNA, specifically the promoter region of genes involved in the synthesis of light-harvesting accessory pigments [19]. However, no homologs of ASRT were identified in other genomes containing a xenorhodopsin gene, suggesting the ASR-ASRT association is a specific feature of *Anabaena (Nostoc) *sp. PCC 7120. Moreover, the identification of ASRT homologs in numerous bacterial and archaeal genomes that lack an ASR (xenorhodopsin) homolog suggests the ASRT protein family is not specific to photosensory signal transduction processes.

The lack of identifiable common transducer elements suggests possible plasticity in the transducer component(s) modulating possible xenorhodopsin-mediated photosensory activity. For example, *Cyanothece sp*. PCC 7424 has genes encoding a two-component regulatory system within the same genomic neighborhood as the xenorhodopsin gene (Figure 3). The two Nanohaloarcheal genomes (*Nanosalina *sp. and *Nanosalinarum *sp.) have genes encoding a putative Na^+^/H^+ ^antiporter system adjacent to the rhodopsin gene. The high sequence identity shared between these transporter sequences along with their conserved genomic location is atypical for these two archaeal genomes, representing different genera, which are generally non-syntenic [7]. It is tempting to speculate that genes in this local region of conservation could be related to rhodopsin function in these organisms.

Despite highly diverse taxonomic origins, the seven species possessing a xenorhodopsin protein share a number of common characteristics, including the absence of flagellar motility, relatively low genomic percent G+C content and isolation from habitats with a high incidence of UV light (Additional File 1: Table S2). The lack of flagellar motility is noteworthy because it eliminates the potential usefulness of previously characterized sensory rhodopsin classes which act by influencing the rotational state of the flagellar motor for phototaxis. The particularly low G+C compositions of *Nanosalina *sp. (43%), *Anabaena (Nostoc) *sp. PCC 7120 (41%), *Cyanothece *sp. PCC 7424 (38%), *Bacillus coahuilensis *(38%) and *H. contractile *(33.6%) are atypical for unicellular inhabitants of high light environments, rendering them especially sensitive to potential UV damage via the formation of thymidine dimers. The isolation of *H. contractile *from deep marine sediments, where light is not a factor, may be an anomaly, since closely related 16S rRNA gene sequences have also been found in high-light solar saltern environments [9].

Consistent with these observations, one intriguing hypothesis is that xenorhodopsins may play a role in pre-emptive photoprotection by inducing light-dependent changes in the expression of photoprotective pigments, a role proposed for the ASR protein due to its photochromic properties [3,6]. Alternatively, these proteins could be linked to expression of DNA repair mechanisms. However, these speculations must be tempered by the caveat that no sensory or ion transport function has yet been experimentally validated for ASR, or any other xenohodopsin protein. Future work on the biochemistry, photochemistry, and molecular genetic characterization of the xenorhodopsin class of proteins will undoubtedly provide fascinating insights into their physiological function in light-induced biological processes.

## Competing interests

The authors declare that they have no competing interests.

## Authors' contributions

All authors conceived the study. JAU and SP performed sequence analysis. PN performed experiments. JAU, SP and EEA wrote the manuscript. All authors read and approved the final manuscript.

## Reviewers' comments

### Reviewer 1

Dr. Michael Y. Galperin, National Center for Biotechnology Information, National Library of Medicine, National Institutes of Health, Bethesda, MD, USA

I agree with the authors' conclusion that Anabaena sensory rhodopsin (ASR) and closely related proteins form a separate family of rhodopsins. However, I believe that the current version of the paper would need a substantial revision to become acceptable for Biology Direct.

The notion that ASR comprises a new type of sensory rhodopsins is not new and should not be presented as such. Spudich and colleagues described the uniqueness of ASR in their early papers [2,16] and unequivocally stated that ASR belongs to a separate family [6]. This does not diminish the contribution of this work, which describes six new members of that family, but the text of the Abstract and the tone of the whole paper must be changed.

#### Author's response

*We thank the reviewer for bringing to our attention these deficiencies in our original summary of previous work recognizing the uniqueness of the ASR protein. We have modified the manuscript to address these issues by changing the title, the abstract, and the interpretational emphasis of our text. We believe these revisions clarify the significance of our findings in discovering that the ASR protein is not a single, isolated anomaly, but rather part of a large, cohesive family of related proteins with an unusual taxonomic distribution. To further emphasize this point, we propose the name "xenorhodopsin" to describe the members of this group, rather than calling them ASR-like (or Sensory Rhodopsin-IV) proteins*.

Although the name "Anabaena sensory rhodopsin" is being widely used in the literature, it is important to note that there has been no experimental proof that this protein actually performs sensory function. Indeed, ASR has been shown not to function as a proton pump and it has been reasoned that it is unlikely to work as a chloride pump. Nevertheless, there remains a distinct possibility that ASR functions as a membrane pump for some other ion, for example, sodium. This proposal is hardly more speculative than the suggestion of the sensory function and is supported by at least three lines of evidence:

1) the adjacency of genes coding for ASR homologs and Na^+^/H^+ ^antiporters, noted by the authors themselves.

2) the observation of Kawanabe *et al. *[14] that a single amino acid change converts ASR into an inward proton pump; and 3) the observation of De Souza *et al. *[20] that so-called ASR transducer is found in a variety of genomes that do not encode ASR and is likely to bind sugars. Further, the previously overlooked absence of the ASRT gene in the complete genome of *Cyanothece *sp. PCC7424 and its recently reported ability to bind DNA [19] strongly suggest that the putative ASR-ASRT signaling cascade is a specific feature of *Anabaena *sp. PCC7120. The authors correctly point out the absence of flagellar motility in the ASR-carrying organisms; this argument, however, is weakened by the chemotactic ability of both *Anabaena *sp. PCC7120 and *Cyanothece *sp. PCC7424, owing to the presence of 3 and 9 methyl-accepting chemotaxis sensors, respectively [21]. In the absence of direct experimental data, the authors should discuss possible alternative functions of the ASR-like family and should be more careful in describing this new rhodopsin family as sensory rhodopsins.

#### Author's response

*We have expanded the text to include a discussion of possible alternative functions for the xenorhodopsin family, including how lack of experimental evidence for ASR sensory function affects interpretation of conserved amino acid sequences, the importance of mutational experiments demonstrating gain of inward proton pumping function, and the apparent species-specific nature of the ASR/ASRT interactions*.

I would also suggest moving the Supplementary Figure S1 (Genomic neighborhood of SR-IV genes) to the main text.

#### Author's response

*The previously presented Supplementary Figure S1 is now *Figure 3*in the main text*.

### Reviewer 2

Dr. Rob Knight, Department of Chemistry and Biochemistry, University of Colorado, Boulder, CO, USA

In this manuscript, the authors analyze a set of microbial rhodopsin sequences (including some that they amplified for this study from an environmental sample), and demonstrate that there is a new clade of sensory rhodopsins that is basal, with high bootstrap support, and that includes sequences from a surprisingly broad phylogenetic range (including one archaeal and three bacterial phyla). This distribution is interesting because previous studies of sensory rhodopsins have found them primarily in the Euryarchaeota and in the Bacteroidetes.

The methods are generally sound except that the taxonomy of the sister groups to the new clade is poorly resolved (i.e. non-significant bootstraps), and it would be reassuring if the split were confirmed using other phylogenetic methods besides likelihood (e.g. distance or Bayesian methods) before the new set of sequences was claimed as distinct.

#### Author's response

*We have supplemented our original phylogenetic analysis with Bayesian and distance-based methods, and find that all agree in supporting identical tree topologies. We have revised *Figure 1*and the text to clarify the fact that the topologies agree and that bootstrap values supporting branches relevant to the new clade are highly significant using all methods*.

Additionally, although the patchy phylogenetic distribution is suggestive of horizontal gene transfer, formal methods (of which several exist) should be used to confirm HGT as opposed to other factors that can lead to gene/species tree incongruence

#### Author's response

*Although many methods of HGT detection have been proposed in the literature, their lack of consistency and potential unreliability in the face of complex, real world data have long been a matter of controversy and debate. Phylogenetic tree incongruency is currently considered the gold standard by which all other HGT prediction methods are judged, and this is the primary technique we have used to reach conclusions presented in the manuscript, which we believe are compelling*.

*To supplement the phylogenetic analyses, we have performed several additional HGT analyses using methods based on DNA signature patterns, included these results as Supplementary Table S1, and expanded discussion of HGT in the text to include interpretation of these additional results*.

## Supplementary Material

Additional File 1 **Supplementary Methods and Tables**. Click here for file

Additional File 2 **Trimmed amino acid alignment file of microbial rhodopsin sequences**. Click here for file

Additional File 3 **SWISS-MODEL 3-dimensional protein structure model of *Nanosalina *sp. xenorhodopsin using ASR as a template**. Click here for file
