# Supplementary material for: Xenorhodopsins, an enigmatic new class of microbial rhodopsins horizontally transferred between archaea and bacteria
Source: Biol Direct. 2011 Oct 10;6:52. doi: 10.1186/1745-6150-6-52 (PMC3198991; doi:10.1186/1745-6150-6-52)
Supplement: Additional File 1 — Supplementary Methods and Tables. [file 1745-6150-6-52-S1.PDF]

Additional File 1

Xenorhodopsins, an enigmatic new class of microbial rhodopsins horizontally transferred between Archaea and Bacteria

Ugalde JA, Podell S, Narasingarao P, Allen EE

Contents

Supplementary Methods

Table S1 - DNA signature properties of xenorhodopsin genes and genomes

Table S2 - G+C content, habitat and motility characteristics of organisms harboring a xenorhodopsin gene

Supplementary Methods

Sample collection and PCR amplification

Surface water samples were collected from a high salinity crystallizer pond at South Bay Salt Works, Chula Vista, California. 20L were passed through a 20 µm Nytex prefilter, followed by sequential filtration through a series of polyethersulfone, 142 mm diameter membrane filters (Pall Corporation) of decreasing porosities (3 µm > 0.8 µm) and finally into a Sterivex filter unit (Milipore Corporation). DNA was extracted from the Sterivex unit following the protocol described in [1]. DNA was amplified using a primer pair designed to maximize PCR product length and specificity, chosen based on aligned xenorhodopsin sequences from *Nanosalina* sp. J07AB43 and *Nanosalinarium* sp. J07AB56:

BOP_F (5'-AGTGGTKTGGTCCGGSCTGA-3')

BOP_R (5'-GAACGGCAGGACRACSAGTGC-3')

The resulting 477 bp PCR product was gel purified, cloned using TOPO TA cloning kit (Invitrogen) into the pCR4 vector, and clones were sequenced (Retrogen, San Diego, CA.)

Homolog identification and phylogenetic analysis

Xenorhodopsin homolog proteins were identified by BLASTP searches against NCBI Genbank [2] and the Integrated Microbial Genomes (IMG) database [3]. One homolog protein, from the Bacillus coahuilensis draft genome, was found to be split into two adjacent, frame-shifted fragments, joined by a 34 nucleotide insertion. These two amino acid fragments were aligned against the ASR protein and manually corrected into a single, full-length composite sequence for further analysis.

All rhodopsin sequences were subsequently aligned with MAFFT version 6.833 [4], using the default parameters. This alignment was manually trimmed to remove hypervariable regions, in particular regions falling within the loops of the rhodopsin structure [5]. The alignment is provided in Additional File 2 (phylip format).

Phylogenetic reconstruction was performed using parameters selected using ProtTest [6], including an LG model with empirical amino acid estimated frequencies, a gamma correction, and an estimated frequency of invariant sites. Phylogenetic analysis was performed using RaxML [7] and MrBayes [8] on the Cipres portal [9]. RaxML maximum likelihood analysis included 1,000 bootstrap replicates. For the Bayesian analysis, a mixed model was used, where the chain was run for 1,000,000 generations and sampled every 1,000th generation.

Genomic DNA signatures

Percent GC and codon usage frequencies were quantified for all predicted coding sequences in each test genome using a custom perl script. Frequencies of all possible codons for each genome were plotted against frequencies of these same codons in each gene of interest to calculate a linear codon correlation coefficient (R^2). Interpolated

Variable Order Motifs were used to identify regions of atypical composition in raw genomic DNA with the program Alien Hunter, version 1.7 [10].

Protein modeling

Homology modeling of the *Nanosalina* sp. rhodopsin was performed using the SWISS-MODEL web server [11] using the automatic modeling mode. The model selected in this mode was the *Anabaena (Nostoc)* sp. rhodopsin, PDB code 1XIO. Visualization, coloring and generation of the movie (Additional File 3) were performed using PyMOL [12].

References

1. Somerville CC, Knight IT, Straube WL, Colwell RR: **Simple, rapid method for direct isolation of nucleic acids from aquatic environments**. *Appl Environ Microbiol* 1989, **55**(3):548-554.
2. Benson DA, Karsch-Mizrachi I, Lipman DJ, Ostell J, Sayers EW: **GenBank**. *Nucleic Acids Res* 2009, **37**(Database issue):D26-31.
3. Markowitz VM, Mavromatis K, Ivanova NN, Chen IM, Chu K, Kyrpides NC: **IMG ER: a system for microbial genome annotation expert review and curation**. *Bioinformatics* 2009, **25**(17):2271-2278.
4. Katoh K, Misawa K, Kuma K, Miyata T: **MAFFT: a novel method for rapid multiple sequence alignment based on fast Fourier transform**. *Nucleic Acids Res* 2002, **30**(14):3059-3066.
5. Ihara K, Umemura T, Katagiri I, Kitajima-Ihara T, Sugiyama Y, Kimura Y, Mukohata Y: **Evolution of the archaeal rhodopsins: evolution rate changes by gene duplication and functional differentiation**. *J Mol Biol* 1999, **285**(1):163-174.
6. Abascal F, Zardoya R, Posada D: **ProtTest: selection of best-fit models of protein evolution**. *Bioinformatics* 2005, **21**(9):2104-2105.
7. Stamatakis A: **RAxML-VI-HPC: maximum likelihood-based phylogenetic analyses with thousands of taxa and mixed models**. *Bioinformatics* 2006, **22**(21):2688-2690.
8. Ronquist F, Huelsenbeck JP: **MrBayes 3: Bayesian phylogenetic inference under mixed models**. *Bioinformatics* 2003, **19**(12):1572-1574.
9. **The CIPRES Portals.** [http://www.phylo.org/sub_sections/portal]
10. Vernikos GS, Parkhill J: **Interpolated variable order motifs for identification of horizontally acquired DNA: revisiting the Salmonella pathogenicity islands**. *Bioinformatics* 2006, **22**(18):2196-2203.
11. Kiefer F, Arnold K, Kunzli M, Bordoli L, Schwede T: **The SWISS-MODEL Repository and associated resources**. *Nucleic Acids Res* 2009, **37**(Database issue):D387-392.
12. Schrodinger, LLC: **The PyMOL Molecular Graphics System, Version 1.3r1**. In.; 2010.

Table S1. DNA signature properties of xenorhodopsin genes and genomes. Gene versus genome GC content and Alien Hunter (Interpolated Variable Order Motif) scores were used to assess evidence for recent horizontal gene transfer. Genome-wide values for percent GC represent mean ± standard deviation, calculated based on coding regions only. R^2 values measure linear correlation between percent usage for each possible codon across all coding regions in the genome, versus percent usage for these same codons in the individual gene. Values suggesting recent gene horizontal transfer are highlighted in bold type. Abbreviation 'nd' indicates Alien Hunter did not detect any candidate features in the region surrounding the gene of interest. Alien Hunter threshold is the minimum feature score considered significant in the context of the test genome.

Organism	Accession number(s)	Prot. length	Gene pct GC	Genome pct GC	Codon R^2 value	Alien Hunter score	Alien Hunter threshold
Anabaena (*Nostoc*) PCC 7120	NP_487205	261	41	42 ± 4.5	0.408	nd	15.0
Cyanothece PCC 7424	YP_002379171.1	264	37	39 ± 4.7	0.531	nd	12.7
Haloplasma contractile	ZP_08554533.1	228	34	33 ± 4.0	0.454	nd	46.1
Hymenobacter roseosalivarius	IMG_2502504230	232	55	57 ± 5.7	0.450	nd	21.4
Nanosalinarum sp. J07NFR56	EGQ40596.1	236	56	56 ± 4.6	0.288	nd	18.7
Nanosalina sp. J07NFR43	EGQ43296.1	228	49	44 ± 4.5	**0.079**	**32.6**	22.4
Bacillus coahuilensis	ZP_03226327.1<br>ZP_03226326.1	216	36	38 ± 6.0	**0.005**	**57.8**	46.1

Table S2. G+C content, habitat and motility characteristics of organisms harboring a xenorhodopsin gene.

Organism	G+C content	Flagellar Motility	Habitat
Nanosalina sp. J07AB43	43%	No	Hypersaline
Nanosalinarum sp. J07AB56	56%	No	Hypersaline
Haloplasma contractile	34%	No	Hypersaline
Bacillus coahuilensis	38%	No	Hypersaline
Hymenobacter roseosalivarus	56%	No	Antarctic soil
Cyanothece sp. PCC 7424	38%	No	Freshwater
Anabaena (*Nostoc*) sp. PCC 7120	41%	No	Freshwater
